# Response to “Oral PrEP for young African women and men”

**DOI:** 10.7448/IAS.19.1.20861

**Published:** 2016-02-25

**Authors:** Connie L Celum, Sinead Delany-Moretlwe, Margaret McConnell, Heidi van Rooyen, Linda-Gail Bekker, Ann Kurth, Elizabeth Bukusi, Chris Desmond, Jennifer Morton, Jared M Baeten

**Affiliations:** 1 Department of Global Health, University of Washington Seattle, WA, USA; 2 Department of Medicine, University of Washington Seattle, WA, USA; 3 Department of Epidemiology, University of Washington Seattle, WA, USA; 4 Wits RHI, University of the Witwatersrand, Johannesburg, South Africa; 5 Department of Global Health and Population, Harvard T.H. Chan School of Public Health, Boston, MA, USA; 6 Human Sciences Research Council, Durban, South Africa; 7 The Desmond Tutu HIV Centre, University of Cape Town, Cape Town, South Africa; 8 School of Nursing, Yale University, New Haven, CT, USA; 9 Kenya Medical Research Institute, Nairobi, Kenya

We appreciate the letter in response to our article, “Rethinking HIV prevention to prepare for oral pre-exposure prophylaxis (PrEP) implementation for young African women.” The authors encouraged the implementation of PrEP among pregnant young women, inquired about programmes addressing oral PrEP implementation for young African men, including as part of combination antiretroviral (ARV)-based prevention for serodiscordant couples in which the HIV-positive partner is offered combination antiretroviral therapy (ART), and the need for evaluation of cost-effectiveness of PrEP in resource-limited settings is highlighted.

We agree that PrEP is an important prevention strategy to offer HIV-negative pregnant women, particularly for women with a known HIV-positive partner. The high viral loads that occur during acute HIV infection significantly increase the risk of HIV transmission to the foetus. PrEP substantially reduces the risk of women becoming HIV-positive, and appears safe during early pregnancy per the Partners PrEP Study [1]. Additional safety data on infant and pregnancy outcomes will be obtained from ongoing PrEP implementation projects among women who are counselled about PrEP effectiveness and available safety data, and choose to continue PrEP throughout their pregnancy. With respect to PrEP delivery, antenatal clinics are an excellent platform for identifying women who would benefit from PrEP.

World Health Organization guidelines recommend PrEP for persons at substantial risk of HIV, operationalized as HIV incidence of 3% or greater [2]. With regards to young African men, local epidemiology should indicate whether men are at substantial risk of HIV, such as in geographic hot spots, transportation hubs and fishing communities. In addition, a key strategy to identify men at substantial risk is through couples counselling and testing, which should be strongly encouraged by all testing and counselling programmes as well as HIV care centres. When HIV-serodiscordant couples are identified through couples testing, HIV treatment should be offered to the HIV-positive partner along with condoms and counselling. We have demonstrated that PrEP offered as a “bridge” until the HIV-positive partner initiates ART is highly acceptable and effective among HIV-serodiscordant couples in Kenya and Uganda, of which two-thirds of the HIV-negative partners taking PrEP were men [3]. This PrEP bridging strategy for HIV-serodiscordant couples is time limited and provides maximal protection until the HIV-positive partner initiates ART and is virally suppressed, regardless of the gender of the HIV-negative partner.

Finally, the cost-effectiveness of PrEP is a critical factor in determining impact, resource allocation and sustainability in low-, middle- and high-income country settings, and needs to be evaluated in non-research delivery settings and for different populations. We have reported on the cost-effectiveness of the PrEP bridging strategy for East African HIV-serodiscordant couples [4]. The cost-effectiveness of PrEP for young African women will be determined by reaching young women at substantial risk of HIV infection, their uptake and adherence to PrEP and delivery costs, all of which will be assessed in ongoing demonstration projects.
